# Pessary Remaining in Vagina for Six Years

**Published:** 1882-02

**Authors:** J. G. Earnest

**Affiliations:** Atlanta, Ga.


### PESSARY REMAINING IN VAGINA FOR SIX YEARS.

By J. G. EARNEST, M.D., Atlanta, Ga.

As there seems to be considerable interest just now in regard to the length of time a pessary may be worn, the following case is contributed to the literature of the subject:

August 19th, 1874, M. T., æt. about 30, mulatto, laundress, applied for relief of symptoms found to be associated with retroversion and hypertrophy of the uterus. The organ was pushed into its normal position and a Hodge lever pessary adjusted to prevent a reproduction of the displacement. She was ordered copious injections of water to the vagina, and instructed to keep the bowels open with a mild aperient.

August 29. She reported her condition much improved, and was told to continue the treatment and report a fortnight later. Nothing more was heard from her until May 20, 1881, when she called to say that she had been suffering from the effects of a copious leucorrhœa set up some months previous, and wanted to know if I thought "that thing" that I gave her several years back had anything to do with it. You can imagine I was surprised when she explained that "that thing" referred to the Hodge ring given her six years and nine months before, and continuously worn ever since without being removed. She stated that she had been practically relieved of all of her troublesome symptoms until two or three months since, when the leucorrhœa returned, and had constantly grown more profuse, until it had now reached a point where it was exhausting her. She also states that when she stands much on her feet or stoops over much, there is an uneasy feeling about the womb, and more or less back ache. An examination revealed the fact that the posterior portion of the ring was almost buried in the tissue of the vagina behind the womb. The womb was anteverted and the neck nearly normal as to size, density, etc. The ring was removed and the vagina examined with a Sims speculum. Pressure of the upper portion of the ring had caused the absorption of the tissue beneath, while the contiguous portion of the mucous membrane had been stimulated to excessive action by the presence of the ring. This deep groove was smooth and free from "granulations" except on the overlapping edges. The walls of the vagina were covered with a discharge resembling "laudable pus"—*i. e.*, thick and creamy. She was ordered copious injections of hot water, and instructed to keep off her feet for a few days.

June 2d. She reported the leucorrhœa much improved, and her general health about as usual. Nothing more has been heard from the case.

